# Supplementary material for: Isolation of Mirificin and Other Bioactive Isoflavone Glycosides from the Kudzu Root Lyophilisate Using Centrifugal Partition and Flash Chromatographic Techniques
Source: Molecules. 2022 Sep 22;27(19):6227. doi: 10.3390/molecules27196227 (PMC9570587; doi:10.3390/molecules27196227)
Supplement: Supplementary file 1 [file molecules-27-06227-s001.zip › molecules-1888768-supplementary.pdf]

molecules

MDPI

Article

Isolation of Mirificin and Other Bioactive Isoflavone Glycosides from the Kudzu Root Lyophilisate Using Centrifugal Partition and Flash Chromatographic Techniques

Magdalena Maciejewska-Turska [1*], Łukasz Pecio [1,2] and Grażyna Zgórka [1]

[1] Department of Pharmacognosy with the Medicinal Plant Garden, Faculty of Pharmacy, Medical University of Lublin, 1 Chodzki Str., 20-093 Lublin, Poland
[2] Department of Biochemistry and Crop Quality, Institute of Soil Science and Plant Cultivation, State Research Institute, 24-100 Pulawy, Poland
* Correspondence: magdalena.maciejewska@umlub.pl

Citation: Lastname, F.; Lastname, F.; Lastname, F. Title. *Molecules* **2022**, *27*, 6227. https://doi.org/10.3390/molecules27196227

Academic Editor: Chia Ming Chang

Received: 12 August 2022
Accepted: 20 September 2022
Published: 22 September 2022

Publisher's Note: MDPI stays neutral with regard to jurisdictional claims in published maps and institutional affiliations.

Copyright: © 2022 by the authors. Licensee MDPI, Basel, Switzerland. This article is an open access article distributed under the terms and conditions of the Creative Commons Attribution (CC BY) license (https://creativecommons.org/licenses/by/4.0/).

Supplementary Materials

Table S1. ^{1}H- and ^{13}C-NMR spectral data obtained for puerarin, 3′-methoxypuerarin, mirificin and daidzin

	**Puerarin ***		**3′-Methoxypuerarin**		**Mirificin**		**Daidzin**	
**No**	δ_H	δ_C	δ_H	δ_C	δ_H	δ_C	δ_H	δ_C
**2**	8.33, s	152.6	8.39, s	152.8	8.32, s	152.5	8.38, s	153.2
**3**		122.5		122.9		122.5		122.3
**4**		174.8		174.8		174.8		174.7
**5**	7.94, d (8.8)	126.2	7.94, d (8.8)	126.2	7.94, d (8.9)	126.2	8.05, d (8.9)	126.9
**6**	6.99, d (8.8)	114.9	6.99, d (8.8)	114.9	6.99, d (8.9)	115.2	7.14, dd (8.9, 2.3)	115.5
**7**		161.0		161.0		160.9		161.4
**8**		112.6		112.5		112.5	7.23, d (2.3)	103.4
**9**		155.9		156.0		156.0		157.0
**10**		116.9		117.0		116.9		118.4
**1′**		123.0		123.0		123.1		123.7
**2′**	7.40, d (8.6)	129.9	7.17, d (2.0)	113.1	7.40, d (8.6)	130.0	7.41, d (8.6)	130.0
**3′**	6.80, d (8.6)	114.9		147.1	6.80, d (8.6)	114.9	6.82, d (8.6)	114.9
**4′**		157.1		146.4		157.1		157.2
**5′**	6.80, d (8.6)	114.9	6.81, d (8.2)	115.1	6.80, d (8.6)	114.9	6.82, d (8.6)	114.9
**6′**	7.40, d (8.6)	129.9	7.04, dd (8.2, 2.0)	121.5	7.40, d (8.6)	130.0	7.41, d (8.6)	130.0
**3′-OCH₃**			3.80, s	55.6				
	**8-*C*-β-Glc*p***		**8-*C*-β-Glc*p***		**8-*C*-β-Glc*p***		**7-*O*-β-Glc*p***	
**1″**	4.82, d (9.8)	73.4	4.82, d (9.9)	73.4	4.81, d (9.7)	73.4	5.10, d (7.4)	100.0
**2″**	4.02, t (9.2)	70.8	4.03, t (9.9)	70.8	4.00, t (9.2)	70.7	3.30, dd (8.9, 7.4)	73.1
**3″**	3.28, t (8.3)	78.7	3.28, t (8.6)	78.7	3.27, t (8.8)	78.6	3.32, t (8.8)	76.5
**4″**	3.22	70.4	3.23	70.5	3.19, t (8.9)	70.5	3.19, t (8.6)	69.6
**5″**	3.24	81.7	3.22	81.8	3.40	80.0	3.46	77.2
**6″**	3.71, dd (11.8, 1.7)<br>3.45, dd (12.0, 5.6)	61.4	3.72, dd (11.8, 1.8)<br>3.45, *br*d (11.8)	61.3	3.92, d (9.2)<br>3.40	68.2	3.72, d (9.7)<br>3.48, dd (11.5, 5.5)	60.6
					**6″-Api*f***			
**1‴**					4.78, d (3.1)	109.0		

**2‴**	3.73, (3.1)	d	75.6
**3‴**			78.7
**4‴**	3.84, (9.4)	d	73.1
	3.57, (9.4)	d	
**5‴**	3.33, (11.4)	d	63.0
	3.29, (11.4)	d	

* NMR spectra recorded at 30° C in DMSO-d_6 with 0.1% TFA, *J* in Hz; overlapping signals are reported without designated multiplicity
